# Crystal structure of (*E*)-2-(*tert*-butyl­amino)-4-(*tert*-butyl­imino)­naphthalen-1(4*H*)-one

**DOI:** 10.1107/S2056989018008514

**Published:** 2018-06-15

**Authors:** Guy Lamoureux, Mónica Alvarado-Rojas, Leslie W. Pineda

**Affiliations:** a Escuela de Química, Universidad de Costa Rica, 2060 San Pedro, San José, Costa Rica; b Centro de Investigación en Productos Naturales (CIPRONA), Universidad de Costa, Rica, 2060 San José, Costa Rica; c Centro de Electroquímica y Energía Química (CELEQ), Universidad de Costa Rica, 2060 San José, Costa Rica

**Keywords:** crystal structure, N—H⋯O hydrogen bonding, intra­molecular hydrogen bonding, naphtho­quinone

## Abstract

The title compound, C_18_H_24_N_2_O, is the first example of a naphtho­quinone imine derivative isolated in the 4-imine/2-amine tautomeric form having bulky alkyl substituents at the N atoms. The mol­ecular conformation is stabilized by an intra­molecular hydrogen bond between the amine and a carbonyl group and by London attraction between the two *tert*-butyl groups. Only van der Waals inter­actions were identified in the crystal packing.

## Abstract 2

The title compound is the first example of a naphtho­quinone imine derivative crystallizing in the 4-imine/2-amine tautomeric form having bulky *tert*-butyl substituents at the N atoms.

## Chemical context   

Naphtho­quinones (naphthalene­diones) form an important part of some pharmacophores in medicinal chemistry (López *et al.*, 2015 ▸). During an exploration of anti­malarial drugs, Fieser (Fieser & Fieser, 1935 ▸) indicated that amino­iminona­phtho­quinones, although difficult to form, had inter­esting medicinal properties. Bullock *et al.* (1969 ▸) provided more efficient ways to synthesize a series of these compounds and further investigated their properties as anti­protozoal agents (Bullock *et al.*, 1970 ▸).

Naturally occurring compounds with a similar structure to these amino­iminona­phtho­quinones are known as hydrolytically stable pigments. Recently, several natural products containing a rigid amino­imino­quinone structure have been isolated and identified: macrophilone A (Zlotkowski *et al.*, 2017 ▸), makaluvamines (Radisky *et al.*, 1993 ▸), isobatzelline (Stierle & Faulkner, 1991 ▸), prianosin (Cheng *et al.*, 1988 ▸), epinardin (D’Ambrosio *et al.*, 1996 ▸), and discorhabdin (Harayama & Kita, 2005 ▸) families. These alkaloid secondary metabolites from marine organisms were found to possess cytotoxic anti­tumor properties. It has been reported that the amino­imino­quinone system may contribute to the cytotoxic activity (LaBarbera & Skibo, 2013 ▸).
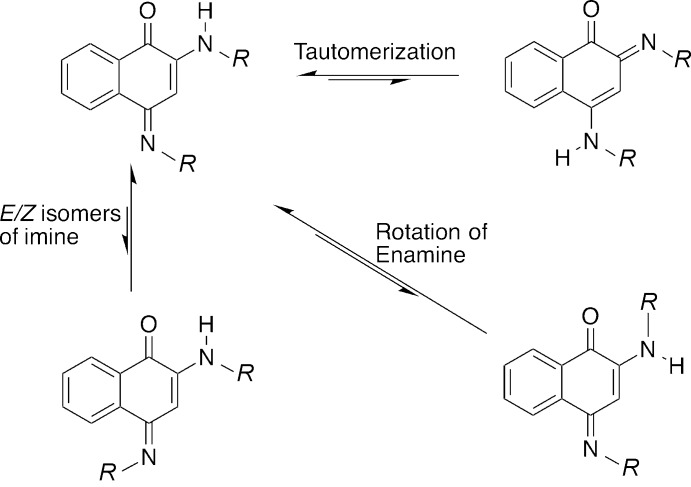


Although the 4-imine/2-amine structure was thought to be the most stable, there is evidence for multiple equilibria of these compounds in solution (see reaction scheme). For example, in the case of the methyl derivative (*R* = Me), NMR evidence at room temperature shows a mixture of tautomers (Bullock *et al.*, 1969 ▸). This equilibrium, and in particular the possibility of tautomers, is important since the biological activity of these compounds depends on which tautomer is more stable (Hatfield *et al.*, 2017 ▸).

As part of our work on the synthesis and properties of naphtho­quinones (Lamoureux *et al.*, 2008 ▸), we isolated the title compound as a minor product and predicted that the 4-imine/2-amine tautomeric form would not form because of the presence of bulky *R* groups. Much to our surprise, (*E*)-2-(*tert*-butyl­amino)-4-(*tert*-butyl­imino)­naphthalen-1(4*H*)-one is the first compound isolated and structurally characterized of this type with a tertiary alkyl group.
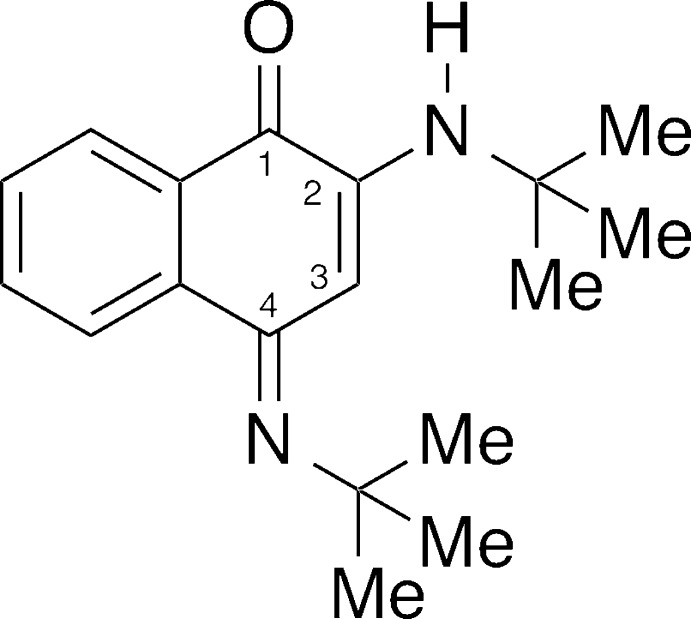


## Structural commentary   

**Figure 1:**
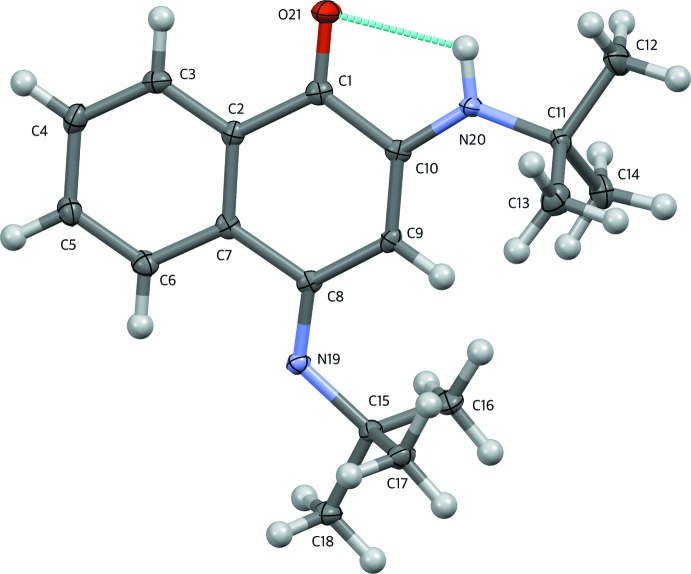
The mol­ecular structure of the title compound with displacement ellipsoids drawn at the 50% probability level. The intra­molecular hydrogen bond is shown as a dotted line.

In the mol­ecule of the title compound (Fig. 1 ▸), the imine C=N bond length at the 4-position [C8—N19 = 1.291 (3) Å] is shorter than the enamine C—N bond length at the 2-position [C10—N20 = 1.353 (3) Å], reflecting the greater double-bond character. The distance between the enamine N-atom and the *t*-butyl C-atom [N20—C11 = 1.476 (3) Å] is slightly shorter than the corresponding bond involving the imino group [N19—C15 = 1.485 (3) Å], possibly as a result of steric compression at the imine. However, the bond angles around the two nitro­gen atoms [C8—N19—C15 = 124.1 (2)°; C10—N20—C11 = 129.2 (2)°] are similar because of the delocalization of π electrons between the two nitro­gen atoms. This system can be considered to be a type of vinyl­ogous amidine (Shriner & Neumann, 1944 ▸), both nitro­gen atoms having a trigonal–planar geometry. The fused imino­quinone ring adopts a flattened envelope conformation, with the flap atom C8 displaced by 0.112 (2) Å from the mean plane through C1/C2/C7/C9/C10, and with the C7—C8—C9 angle of 116.9 (2)° showing the largest deviation from the ideal value of 120°.

The title compound possesses an intra­molecular hydrogen bond between the imine N—H and carbonyl groups (Table 1 ▸), forming a ring with *S*(5) graph-set motif. The distance between the donor H atom and the acceptor carbonyl oxygen atom of 2.20 Å is shorter than expected as a result of the bulkiness of the *tert*-butyl group (*vide infra*). These *tert*-butyl groups also shield the nitro­gen atoms and provide a hydro­phobic environment on the side of the naphthalen-1-one ring system. The shortest C⋯C separations between carbon atoms of the *tert*-butyl groups are in the range 4.228 (4)–4.825 (4) Å, bringing them within distance of London attraction (Wagner & Schreiner, 2015 ▸).

## Supra­molecular features   

**Figure 2:**
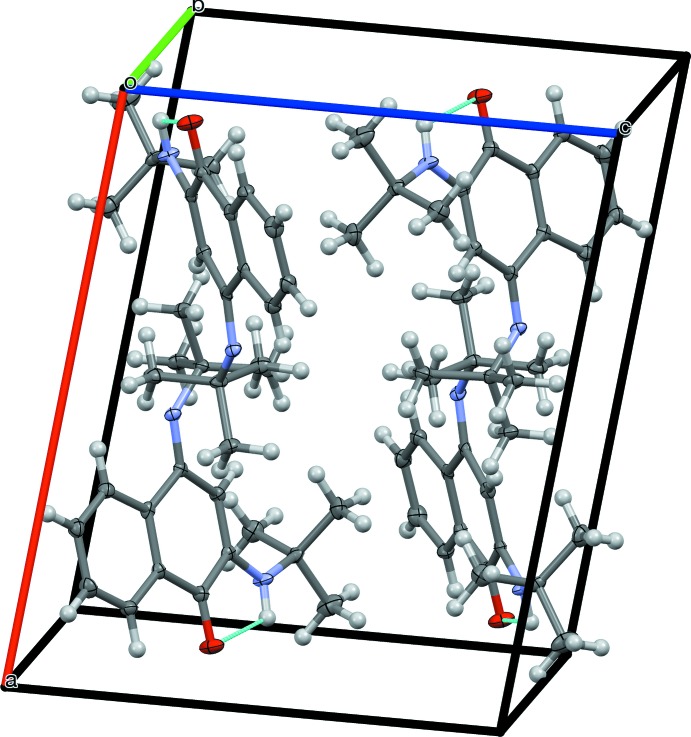
Unit-cell contents of the title compound. Intra­molecular hydrogen bonds are shown in turquoise.

In the crystal structure of the title compound (Fig. 2 ▸), the *tert*-butyl groups are oriented toward the centre of the unit cell. There are no inter­molecular hydrogen bonds, as seen in a similar structure with *n*-butyl groups (see below); the *tert*-butyl groups are shielding the nitro­gen atoms and preventing close approach of the supra­molecular donors and acceptors. There are no π–π stacking inter­actions present, the aromatic rings being separated by more than 6 Å.

## Database survey   

A search of the Cambridge Structural Database (Version 5.39, update February 2018; Groom *et al.*, 2016 ▸) for the substructure 2-(alkyl­amino)-4-(alkyl­imino)­naphthalen-1(4*H*)-one yielded three hits. Two of the structures, ESOFID (Schweinfurth *et al.*, 2016 ▸) and UDAZEF (Singh *et al.*, 2007 ▸) have aromatic amines (aniline or substituted aniline) as the amine moiety. Only one structure, UDAZIJ (Singh *et al.*, 2007 ▸), has an aliphatic primary amine (*n*-butyl­amine) at positions 2 and 4. The structure of UDAZIJ is noteworthy because the intra­molecular N—H⋯O separation of 2.34 Å is much longer than that observed in the title compound, and because in the crystal lattice a dimeric assembly forms, held together by pairs of inter­molecular hydrogen-bonding inter­actions between the N—H and carbonyl groups of centrosymmetrically -related mol­ecules.

## Synthesis and crystallization   

The synthesis of the title compound was based on a new procedure (complete publication in progress). 192 mg (1.00 mmol) of 4-chloro­naphthalene-1,2-dione and 211 µL (2.00 mmol, 2 equiv.) of *tert*-butyl­amine were dissolved in *tert*-amyl alcohol (3.0 mL). This solution was stirred at 383 K under a nitro­gen atmosphere for 2 h. After being allowed to cool to room temperature, the green–brownish solution (originally yellow) was diluted with saline water (30 mL) and extracted with ethyl acetate (3 × 20 mL). The combined organic layers were dried over Na_2_SO_4_, filtered, and then concentrated under reduced pressure. The crude brown-dark solid material (249 mg) was separated by silica gel column chromatography using ethyl acetate as eluent to obtain the title compound as secondary product in the form of a dark-brown oily solid (119 mg). The compound was further purified by column chromatography over silica gel with gradient solvent elution [100% di­chloro­methane (CH_2_Cl_2_) and then 100% methyl *tert*-butyl ether (C_5_H_12_O)], and the fractions were dried under vacuum to yield 14 mg of the pure product (5% yield) as a yellow oily solid. Part of the purified product was redissolved in methanol with a few drops of water and placed at room temperature for slow evaporation. After several days, yellow crystal plates suitable for X-ray analysis were obtained. M.p. 377–388 K using a Fisher–Johns melting-point apparatus with calibrated thermometer. ^1^H NMR (600 MHz, CDCl_3_) δ 8.46–8.48 (*dd*, *J* = 7.8, 1.3 Hz, 1 H), 8.09–8.12 (*dd*, *J* = 7.8, 1.3 Hz, 1 H), 7.61–7.64 (*td*, *J* = 7.8, 1.3 Hz, 1 H), 7.48–7.52 (*td*, *J* = 7.8, 1.2 Hz, 1 H), 6.36 (*s*, 1 H), 5.53 (*br s*, 1 H), 1.56 (*s*, 9 H), 1.47 (*s*, 9 H).

## Refinement   

Crystal data, data collection and structure refinement details are summarized in Table 2 ▸. All hydrogen atoms are placed in calculated positions with N—H = 0.88 Å, C—H = 0.95–0.98 Å, and with *U*_iso_(H) = 1.2*U*_eq_(C, N) or 1.5*U*_eq_(C) for methyl H atoms. A rotating model was used for the methyl groups.

## Supplementary Material

Crystal structure: contains datablock(s) global, I. DOI: 10.1107/S2056989018008514/rz5237sup1.cif

Structure factors: contains datablock(s) I. DOI: 10.1107/S2056989018008514/rz5237Isup2.hkl

Supporting information file. DOI: 10.1107/S2056989018008514/rz5237Isup3.cml

CCDC reference: 1842160

Additional supporting information:  crystallographic information; 3D view; checkCIF report

## Figures and Tables

**Table 1:** Hydrogen-bond geometry (Å, °)

*D*—H⋯*A*	*D*—H	H⋯*A*	*D*⋯*A*	*D*—H⋯*A*
N20—H20⋯O21	0.88	2.20	2.629 (3)	109

**Table 2:** Experimental details

Crystal data	
Chemical formula	C_18_H_24_N_2_O
*M*_r_	284.39
Crystal system, space group	Monoclinic, *P*2_1_/*c*
Temperature (K)	100
*a*, *b*, *c* (Å)	14.2792 (18), 9.8936 (13), 11.4978 (13)
β (°)	97.539 (4)
*V* (Å^3^)	1610.3 (3)
*Z*	4
Radiation type	Mo *K*α
μ (mm^−1^)	0.07
Crystal size (mm)	0.50 × 0.50 × 0.10

Data collection	
Diffractometer	Bruker D8 Venture
Absorption correction	Multi-scan (*SADABS*; Bruker, 2015 ▸)
*T*_min_, *T*_max_	0.688, 0.746
No. of measured, independent and observed [*I* > 2σ(*I*)] reflections	42010, 3732, 1927
*R*_int_	0.213
(sin θ/λ)_max_ (Å^−1^)	0.652

Refinement	
*R*[*F* ^2^ > 2σ(*F* ^2^)], *wR*(*F* ^2^), *S*	0.079, 0.143, 1.01
No. of reflections	3732
No. of parameters	196
H-atom treatment	H-atom parameters constrained
Δρ_max_, Δρ_min_ (e Å^−3^)	0.27, −0.29

Computer programs: *APEX3* (Bruker, 2015 ▸), *SAINT* (Bruker, 2015 ▸), *SHELXT* (Sheldrick, 2015*a* ▸), *SHELXL2014* (Sheldrick, 2015*b* ▸), *Mercury* (Macrae *et al.*, 2006 ▸).

## supplementary crystallographic information

### Crystal data

**Table:**

C_18_H_24_N_2_O	*F*(000) = 616
*M**_r_* = 284.39	*D*_x_ = 1.173 Mg m^−^^3^
Monoclinic, *P*2_1_/*c*	Mo *K*α radiation, λ = 0.71073 Å
*a* = 14.2792 (18) Å	Cell parameters from 68 reflections
*b* = 9.8936 (13) Å	θ = 8.7–51.9°
*c* = 11.4978 (13) Å	µ = 0.07 mm^−^^1^
β = 97.539 (4)°	*T* = 100 K
*V* = 1610.3 (3) Å^3^	Plate, clear light yellow
*Z* = 4	0.50 × 0.50 × 0.10 mm

### Data collection

**Table:**

Bruker D8 Venture diffractometer	3732 independent reflections
Radiation source: Incoatec Microsource	1927 reflections with *I* > 2σ(*I*)
Mirrors monochromator	*R*_int_ = 0.213
Detector resolution: 10.4167 pixels mm^-1^	θ_max_ = 27.6°, θ_min_ = 2.5°
ω scans	*h* = −18→18
Absorption correction: multi-scan (SADABS; Bruker, 2015)	*k* = −12→12
*T*_min_ = 0.688, *T*_max_ = 0.746	*l* = −14→14
42010 measured reflections	

### Refinement

**Table:**

Refinement on *F*^2^	Primary atom site location: structure-invariant direct methods
Least-squares matrix: full	Secondary atom site location: difference Fourier map
*R*[*F*^2^ > 2σ(*F*^2^)] = 0.079	Hydrogen site location: inferred from neighbouring sites
*wR*(*F*^2^) = 0.143	H-atom parameters constrained
*S* = 1.01	*w* = 1/[σ^2^(*F*_o_^2^) + (0.0503*P*)^2^ + 0.5846*P*] where *P* = (*F*_o_^2^ + 2*F*_c_^2^)/3
3732 reflections	(Δ/σ)_max_ < 0.001
196 parameters	Δρ_max_ = 0.27 e Å^−^^3^
0 restraints	Δρ_min_ = −0.29 e Å^−^^3^

### Special details

**Table:**

Geometry. All esds (except the esd in the dihedral angle between two l.s. planes) are estimated using the full covariance matrix. The cell esds are taken into account individually in the estimation of esds in distances, angles and torsion angles; correlations between esds in cell parameters are only used when they are defined by crystal symmetry. An approximate (isotropic) treatment of cell esds is used for estimating esds involving l.s. planes.

### Fractional atomic coordinates and isotropic or equivalent isotropic displacement parameters (Å^2^)

**Table:**

	*x*	*y*	*z*	*U*_iso_*/*U*_eq_	
O21	0.96284 (12)	0.43511 (18)	0.34929 (16)	0.0228 (5)	
N19	0.59245 (14)	0.4688 (2)	0.17726 (17)	0.0128 (5)	
N20	0.87654 (14)	0.6571 (2)	0.40230 (18)	0.0158 (5)	
H20	0.9346	0.6315	0.4279	0.019*	
C1	0.88028 (17)	0.4461 (3)	0.3057 (2)	0.0143 (6)	
C2	0.83366 (17)	0.3446 (2)	0.2234 (2)	0.0127 (6)	
C3	0.88527 (18)	0.2349 (3)	0.1908 (2)	0.0166 (6)	
H3	0.9494	0.2242	0.224	0.02*	
C4	0.84444 (18)	0.1415 (3)	0.1110 (2)	0.0198 (7)	
H4	0.8799	0.0664	0.0895	0.024*	
C5	0.75077 (18)	0.1583 (3)	0.0622 (2)	0.0189 (6)	
H5	0.7226	0.0953	0.0058	0.023*	
C6	0.69833 (18)	0.2659 (3)	0.0950 (2)	0.0166 (6)	
H6	0.6342	0.2755	0.0617	0.02*	
C7	0.73876 (17)	0.3605 (2)	0.1764 (2)	0.0116 (6)	
C8	0.68184 (17)	0.4750 (2)	0.2136 (2)	0.0114 (6)	
C9	0.73246 (17)	0.5786 (2)	0.2858 (2)	0.0126 (6)	
H9	0.6993	0.6582	0.302	0.015*	
C10	0.82484 (16)	0.5677 (2)	0.3316 (2)	0.0116 (6)	
C11	0.84947 (17)	0.7914 (3)	0.4426 (2)	0.0152 (6)	
C12	0.93401 (19)	0.8415 (3)	0.5258 (3)	0.0283 (7)	
H12A	0.9899	0.8441	0.4845	0.042*	
H12B	0.9457	0.7801	0.593	0.042*	
H12C	0.921	0.9324	0.5533	0.042*	
C13	0.83000 (19)	0.8877 (3)	0.3390 (2)	0.0227 (7)	
H13A	0.8865	0.8949	0.2993	0.034*	
H13B	0.8137	0.9771	0.3671	0.034*	
H13C	0.7773	0.8531	0.2839	0.034*	
C14	0.76476 (18)	0.7821 (3)	0.5099 (2)	0.0232 (7)	
H14A	0.7087	0.7557	0.4562	0.035*	
H14B	0.7538	0.8703	0.5446	0.035*	
H14C	0.7772	0.7144	0.5722	0.035*	
C15	0.52276 (17)	0.5718 (3)	0.2041 (2)	0.0142 (6)	
C16	0.52078 (18)	0.5907 (3)	0.3365 (2)	0.0197 (6)	
H16A	0.4676	0.6492	0.3491	0.03*	
H16B	0.58	0.6326	0.3717	0.03*	
H16C	0.5135	0.5026	0.3731	0.03*	
C17	0.53815 (18)	0.7053 (3)	0.1418 (2)	0.0189 (6)	
H17A	0.5372	0.6887	0.0576	0.028*	
H17B	0.5994	0.7437	0.174	0.028*	
H17C	0.4877	0.7689	0.1539	0.028*	
C18	0.42656 (17)	0.5160 (3)	0.1510 (2)	0.0187 (6)	
H18A	0.4251	0.5067	0.0659	0.028*	
H18B	0.3766	0.5783	0.1677	0.028*	
H18C	0.4164	0.4274	0.1854	0.028*	

### Atomic displacement parameters (Å^2^)

**Table:**

	*U*^11^	*U*^22^	*U*^33^	*U*^12^	*U*^13^	*U*^23^
O21	0.0141 (11)	0.0190 (11)	0.0329 (12)	0.0019 (8)	−0.0057 (9)	−0.0047 (9)
N19	0.0124 (12)	0.0116 (11)	0.0140 (12)	0.0017 (9)	0.0003 (9)	0.0013 (10)
N20	0.0111 (12)	0.0129 (12)	0.0215 (13)	0.0013 (9)	−0.0049 (10)	−0.0031 (10)
C1	0.0143 (15)	0.0139 (15)	0.0150 (14)	−0.0006 (11)	0.0027 (12)	0.0023 (12)
C2	0.0155 (14)	0.0113 (14)	0.0118 (14)	0.0002 (12)	0.0036 (11)	0.0007 (11)
C3	0.0131 (14)	0.0200 (15)	0.0165 (15)	0.0021 (12)	0.0012 (11)	0.0025 (13)
C4	0.0247 (16)	0.0149 (15)	0.0204 (15)	0.0039 (12)	0.0052 (13)	−0.0018 (12)
C5	0.0209 (16)	0.0197 (16)	0.0163 (15)	−0.0018 (13)	0.0036 (12)	−0.0043 (12)
C6	0.0151 (14)	0.0183 (15)	0.0168 (15)	−0.0018 (12)	0.0029 (11)	−0.0007 (13)
C7	0.0157 (14)	0.0085 (14)	0.0108 (14)	−0.0026 (11)	0.0024 (11)	0.0036 (11)
C8	0.0130 (14)	0.0102 (14)	0.0112 (14)	0.0002 (11)	0.0029 (11)	0.0045 (11)
C9	0.0148 (14)	0.0096 (13)	0.0138 (14)	0.0015 (11)	0.0034 (11)	0.0013 (11)
C10	0.0123 (14)	0.0119 (14)	0.0106 (13)	−0.0013 (11)	0.0017 (11)	0.0039 (11)
C11	0.0158 (14)	0.0133 (14)	0.0154 (15)	−0.0005 (12)	−0.0025 (11)	−0.0036 (11)
C12	0.0288 (18)	0.0173 (16)	0.0353 (18)	0.0003 (13)	−0.0085 (14)	−0.0080 (14)
C13	0.0268 (16)	0.0162 (15)	0.0248 (16)	0.0000 (13)	0.0025 (13)	0.0016 (13)
C14	0.0277 (17)	0.0252 (16)	0.0169 (15)	0.0006 (14)	0.0033 (12)	−0.0065 (13)
C15	0.0110 (13)	0.0149 (14)	0.0166 (14)	0.0010 (11)	0.0014 (11)	−0.0029 (12)
C16	0.0134 (14)	0.0232 (16)	0.0227 (16)	0.0018 (12)	0.0032 (12)	−0.0034 (13)
C17	0.0166 (15)	0.0151 (15)	0.0241 (15)	0.0033 (12)	−0.0005 (12)	0.0019 (13)
C18	0.0133 (14)	0.0191 (15)	0.0228 (16)	0.0015 (12)	−0.0013 (12)	−0.0016 (13)

### Geometric parameters (Å, º)

**Table:**

O21—C1	1.224 (3)	C11—C13	1.522 (4)
N19—C8	1.291 (3)	C11—C14	1.522 (4)
N19—C15	1.485 (3)	C12—H12A	0.98
N20—C10	1.353 (3)	C12—H12B	0.98
N20—C11	1.476 (3)	C12—H12C	0.98
N20—H20	0.88	C13—H13A	0.98
C1—C2	1.477 (3)	C13—H13B	0.98
C1—C10	1.491 (3)	C13—H13C	0.98
C2—C3	1.391 (3)	C14—H14A	0.98
C2—C7	1.400 (3)	C14—H14B	0.98
C3—C4	1.376 (3)	C14—H14C	0.98
C3—H3	0.95	C15—C18	1.531 (3)
C4—C5	1.391 (3)	C15—C17	1.533 (3)
C4—H4	0.95	C15—C16	1.537 (3)
C5—C6	1.382 (3)	C16—H16A	0.98
C5—H5	0.95	C16—H16B	0.98
C6—C7	1.394 (3)	C16—H16C	0.98
C6—H6	0.95	C17—H17A	0.98
C7—C8	1.489 (3)	C17—H17B	0.98
C8—C9	1.451 (3)	C17—H17C	0.98
C9—C10	1.359 (3)	C18—H18A	0.98
C9—H9	0.95	C18—H18B	0.98
C11—C12	1.521 (3)	C18—H18C	0.98
			
C8—N19—C15	124.1 (2)	C11—C12—H12B	109.5
C10—N20—C11	129.2 (2)	H12A—C12—H12B	109.5
C10—N20—H20	115.4	C11—C12—H12C	109.5
C11—N20—H20	115.4	H12A—C12—H12C	109.5
O21—C1—C2	122.1 (2)	H12B—C12—H12C	109.5
O21—C1—C10	119.9 (2)	C11—C13—H13A	109.5
C2—C1—C10	118.0 (2)	C11—C13—H13B	109.5
C3—C2—C7	120.2 (2)	H13A—C13—H13B	109.5
C3—C2—C1	119.5 (2)	C11—C13—H13C	109.5
C7—C2—C1	120.2 (2)	H13A—C13—H13C	109.5
C4—C3—C2	120.7 (2)	H13B—C13—H13C	109.5
C4—C3—H3	119.7	C11—C14—H14A	109.5
C2—C3—H3	119.7	C11—C14—H14B	109.5
C3—C4—C5	119.3 (2)	H14A—C14—H14B	109.5
C3—C4—H4	120.3	C11—C14—H14C	109.5
C5—C4—H4	120.3	H14A—C14—H14C	109.5
C6—C5—C4	120.6 (3)	H14B—C14—H14C	109.5
C6—C5—H5	119.7	N19—C15—C18	105.09 (19)
C4—C5—H5	119.7	N19—C15—C17	110.7 (2)
C5—C6—C7	120.6 (2)	C18—C15—C17	107.5 (2)
C5—C6—H6	119.7	N19—C15—C16	112.9 (2)
C7—C6—H6	119.7	C18—C15—C16	107.9 (2)
C6—C7—C2	118.6 (2)	C17—C15—C16	112.3 (2)
C6—C7—C8	120.6 (2)	C15—C16—H16A	109.5
C2—C7—C8	120.8 (2)	C15—C16—H16B	109.5
N19—C8—C9	128.0 (2)	H16A—C16—H16B	109.5
N19—C8—C7	115.1 (2)	C15—C16—H16C	109.5
C9—C8—C7	116.9 (2)	H16A—C16—H16C	109.5
C10—C9—C8	123.4 (2)	H16B—C16—H16C	109.5
C10—C9—H9	118.3	C15—C17—H17A	109.5
C8—C9—H9	118.3	C15—C17—H17B	109.5
N20—C10—C9	127.3 (2)	H17A—C17—H17B	109.5
N20—C10—C1	112.7 (2)	C15—C17—H17C	109.5
C9—C10—C1	120.0 (2)	H17A—C17—H17C	109.5
N20—C11—C12	105.8 (2)	H17B—C17—H17C	109.5
N20—C11—C13	110.3 (2)	C15—C18—H18A	109.5
C12—C11—C13	109.7 (2)	C15—C18—H18B	109.5
N20—C11—C14	111.3 (2)	H18A—C18—H18B	109.5
C12—C11—C14	108.5 (2)	C15—C18—H18C	109.5
C13—C11—C14	111.1 (2)	H18A—C18—H18C	109.5
C11—C12—H12A	109.5	H18B—C18—H18C	109.5
			
O21—C1—C2—C3	−2.0 (4)	C6—C7—C8—C9	−171.7 (2)
C10—C1—C2—C3	175.5 (2)	C2—C7—C8—C9	9.0 (3)
O21—C1—C2—C7	179.4 (2)	N19—C8—C9—C10	171.5 (2)
C10—C1—C2—C7	−3.1 (3)	C7—C8—C9—C10	−8.2 (3)
C7—C2—C3—C4	0.9 (4)	C11—N20—C10—C9	−4.0 (4)
C1—C2—C3—C4	−177.7 (2)	C11—N20—C10—C1	176.4 (2)
C2—C3—C4—C5	0.4 (4)	C8—C9—C10—N20	−177.9 (2)
C3—C4—C5—C6	−1.3 (4)	C8—C9—C10—C1	1.7 (4)
C4—C5—C6—C7	0.9 (4)	O21—C1—C10—N20	1.4 (3)
C5—C6—C7—C2	0.5 (4)	C2—C1—C10—N20	−176.2 (2)
C5—C6—C7—C8	−178.8 (2)	O21—C1—C10—C9	−178.3 (2)
C3—C2—C7—C6	−1.4 (4)	C2—C1—C10—C9	4.1 (3)
C1—C2—C7—C6	177.2 (2)	C10—N20—C11—C12	176.7 (2)
C3—C2—C7—C8	177.9 (2)	C10—N20—C11—C13	−64.8 (3)
C1—C2—C7—C8	−3.5 (3)	C10—N20—C11—C14	59.0 (3)
C15—N19—C8—C9	0.2 (4)	C8—N19—C15—C18	−175.8 (2)
C15—N19—C8—C7	179.9 (2)	C8—N19—C15—C17	68.5 (3)
C6—C7—C8—N19	8.6 (3)	C8—N19—C15—C16	−58.5 (3)
C2—C7—C8—N19	−170.7 (2)		

### Hydrogen-bond geometry (Å, º)

**Table:**

*D*—H···*A*	*D*—H	H···*A*	*D*···*A*	*D*—H···*A*
N20—H20···O21	0.88	2.20	2.629 (3)	109

## References

[bb1] Bruker (2015). *APEX3*, *SAINT* and *SADABS*. Bruker AXS Inc., Madison, Wisconsin, USA.

[bb2] Bullock, F. J., Tweedie, J. F. & McRitchie, D. D. (1969). *J. Chem. Soc. C*, pp. 1799–1803.

[bb3] Bullock, F. J., Tweedie, J. F., McRitchie, D. D. & Tucker, M. A. (1970). *J. Med. Chem.* **13**, 550–552. 10.1021/jm00297a051 5441142

[bb4] Cheng, J. F., Ohizumi, Y., Walchli, M. R., Nakamura, H., Hirata, Y., Sasaki, T. & Kobayashi, J. (1988). *J. Org. Chem.* **53**, 4621–4624.

[bb5] D’Ambrosio, M., Guerriero, A., Chiasera, G., Pietra, F. & Tatò, M. (1996). *Tetrahedron*, **52**, 8899–8906.

[bb6] Fieser, L. F. & Fieser, M. (1935). *J. Am. Chem. Soc.* **57**, 491–494.

[bb7] Groom, C. R., Bruno, I. J., Lightfoot, M. P. & Ward, S. C. (2016). *Acta Cryst.* B**72**, 171–179. 10.1107/S2052520616003954 PMC4822653 27048719

[bb8] Harayama, Y. & Kita, Y. (2005). *Curr. Org. Chem.* **9**, 1567–1588.

[bb9] Hatfield, M. J., Chen, J., Fratt, E. M., Chi, L., Bollinger, J. C., Binder, R. J., Bowling, J., Hyatt, J. L., Scarborough, J., Jeffries, C. & Potter, P. M. (2017). *J. Med. Chem.* **60**, 1568–1579. 10.1021/acs.jmedchem.6b01849 PMC5556379 28112927

[bb10] LaBarbera, D. V. & Skibo, E. B. (2013). *J. Org. Chem.* **78**, 11887–11895. 10.1021/jo401927n 24228868

[bb11] Lamoureux, G., Perez, A. L., Araya, M. & Agüero, C. (2008). *J. Phys. Org. Chem.* **21**, 1022–1028.

[bb12] López, J., de la Cruz, F., Alcaraz, Y., Delgado, F. & Vázquez, M. A. (2015). *Med. Chem. Res.* **24**, 3599–3620.

[bb13] Macrae, C. F., Edgington, P. R., McCabe, P., Pidcock, E., Shields, G. P., Taylor, R., Towler, M. & van de Streek, J. (2006). *J. Appl. Cryst.* **39**, 453–457.

[bb14] Radisky, D. C., Radisky, E. S., Barrows, L. R., Copp, B. R., Kramer, R. A. & Ireland, C. M. (1993). *J. Am. Chem. Soc.* **115**, 1632–1638.

[bb15] Schweinfurth, D., Mazzolini, M., Neshchadin, D., Hoyer, C., Geier, R., Gatterer, K., Trapp, N., Gescheidt, G. & Diederich, F. (2016). *Chem. Eur. J.* **22**, 7152–7157. 10.1002/chem.201600611 27106784

[bb16] Sheldrick, G. M. (2015*a*). *Acta Cryst.* A**71**, 3–8.

[bb17] Sheldrick, G. M. (2015*b*). *Acta Cryst.* C**71**, 3–8.

[bb18] Shriner, R. L. & Neumann, F. W. (1944). *Chem. Rev.* **35**, 351–425.

[bb19] Singh, M. W., Karmakar, A., Barooah, N. & Baruah, J. B. (2007). *Beilstein J. Org. Chem.* **3**, No. 10. 10.1186/1860-5397-3-10 PMC1838918 17331232

[bb20] Stierle, D. B. & Faulkner, D. J. (1991). *J. Nat. Prod.* **54**, 1131–1133. 10.1021/np50076a039 1791477

[bb21] Wagner, J. P. & Schreiner, P. R. (2015). *Angew. Chem. Int. Ed.* **54**, 12274–12296. 10.1002/anie.201503476 26262562

[bb22] Zlotkowski, K., Hewitt, W. M., Yan, P., Bokesch, H. R., Peach, M. L., Nicklaus, M. C., O’Keefe, B. R., McMahon, J. B., Gustafson, K. R. & Schneekloth, J. S. Jr (2017). *Org. Lett.* **19**, 1726–1729. 10.1021/acs.orglett.7b00496 PMC6318790 28345939

